# Exploring the Relationship of Smartphone Addiction on Attention Deficit, Hyperactivity Symptoms, and Sleep Quality Among University Students: A Cross‐Sectional Study

**DOI:** 10.1002/brb3.70137

**Published:** 2024-11-22

**Authors:** Ibrahim Zeyrek, Muhammed Fatih Tabara, Mahmut Çakan

**Affiliations:** ^1^ Department of Child and Adolescent Psychiatry Bingol Obstetrics and Gynecology and Child Diseases Hospital Bingol Turkey; ^2^ Department of Psychiatry Firat University School of Medicine Elazig Turkey; ^3^ Bingol Pılot Unıversıty Coordınatıon Center Bingol University Bingol Turkey

**Keywords:** addiction, attention, hyperactivity, sleep quality, smartphone

## Abstract

**Background:**

The prevalence of smartphone usage is steadily rising, leading to the potential development of addiction due to problematic use. This study examined the relationship between smartphone addiction, self‐perceived attention deficit and hyperactivity symptoms, and sleep quality among 443 university students at Bingöl University.

**Methods:**

Participants completed several questionnaires, including the Smartphone Addiction Scale‐Short Version, the Pittsburgh Sleep Quality Index, and the Adult ADHD Self‐Report Scale.

**Results:**

This study examined 443 participants, with a mean age of 20.97 ± 3.29, of whom 72.7% (*n* = 322) were female. Results showed that the majority of participants primarily used smartphones (94.8%, *n* = 420) for social media browsing (49.9%, *n* = 221). Factors such as smoking, preference for smartphone screens, and prolonged screen exposure significantly affected sleep quality. Smartphone addiction rates were notable, with 50.4% (*n* = 61) of males and 47.2% (*n* = 152) of females being affected; this addiction was associated with poorer sleep quality. Correlations were found between age, sleep duration, and scores on smartphone addiction, sleep quality, and attention deficit scales. Linear regression analysis revealed that age, attention deficit scores, and sleep quality scores significantly influenced levels of smartphone addiction.

**Conclusions:**

These findings contribute valuable insights into the impact of smartphone addiction on attention and sleep in university students.

## Graphical abstract

Smartphone addiction is common among university students. In addition, students with smartphone addiction are more likely to show symptoms of ADHD. Smoking, a preference for smartphones as a screen, and long daily screen exposure impair sleep quality.

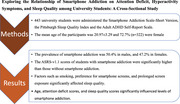

## 1 Introduction

The utilization of digital devices among young adults, particularly university students, is pervasive for both social and educational purposes. Consequently, smartphones, televisions, computers, and tablets have become indispensable components of their lives (Chen and Peng 2008). On a global scale, the number of individuals with smartphone data plans currently exceeds 6 billion, and projections estimate this figure to surpass 7.5 billion by 2026 (Statista 2021). The prevalence of mobile phone ownership among students is remarkably high, as evidenced by a study where 99.7% of respondents reported owning one or more mobile phones (Chaputula and Mutula 2018). Research indicates that the occurrence of mobile phone dependence resulting from Internet addiction (IA) is widespread among adolescents (Mak et al. 2014).

Smartphones have the same functions as computers, but they are much smaller and wireless. This makes them more convenient for young people, who have more access to smartphones than other devices (Brodersen, Hammami, and Katapally 2022). However, this convenience also comes with some risks. Many studies have looked at how screen time affects teenagers. For example, one study found that college students spend a lot of time on electronic devices, at least 3 h a day, on one or more devices (Montagni, Guichard, and Kurth 2016).

Smartphone addiction is a problem that arises from its widespread use and many functions (Kwon, Kim, et al., 2013). It has a lot in common with IA (H. Kim 2013). However, it also has some unique features, such as mobility, real‐time Internet access, and direct communication with smartphones (Kwon, Lee, et al., 2013). The longer and more often people use smartphones, the worse their addiction gets (Lee et al. 2014). There is no official way to diagnose smartphone addiction. However, it has been described as the overuse of smartphones that disrupts users' daily lives (Demirci, Akgönül, and Akpinar 2015).

Attention‐deficit hyperactivity disorder (ADHD) is a neurodevelopmental disorder typically diagnosed in childhood and can persist into adulthood. It is characterized by symptoms of hyperactivity, impulsivity, and inattentiveness, which can impact social, academic, and occupational performance and development, either individually or in combination (Batstra and Frances 2012). The prevalence of ADHD during childhood is estimated to be around 5%–7% (Thomas et al. 2015), while in adulthood, it ranges from 2% to 3% (Fayyad et al. 2017). Research indicates that young individuals with ADHD may display unhealthy behaviors such as excessive screen time, limited engagement in physical activities, and poor dietary habits (Must et al. 2015). Furthermore, there is a notable association between ADHD symptoms among university students and IA (Dalbudak and Evren 2014; D. Kim et al. 2013).

According to a review paper, there is evidence suggesting a connection between smartphone usage and negative effects on mental health (Thomée 2018). Moreover, a meta‐analytic review specifically emphasizes the relationship between smartphone use and stress and anxiety (D. Kim et al. 2013). Another systematic review confirms the correlations between problematic smartphone use and the severity of depression (Elhai et al. 2017). In the United States and other countries, watching TV and using computers are common sedentary activities. People who spend more than 4 h engaging in screen time, which includes TV watching and computer use, have an increased likelihood of developing depression (de Wit et al. 2011).

The impact of economic and technological progress on young students is evident in the increased amount of time they spend in front of screens. Numerous health problems, including cardiovascular disease, mental health issues, disrupted sleep quality, and decreased academic performance, have consistently been associated with excessive screen exposure among young individuals (Wu et al. 2015).

The complete impact of screen time on young adults remains uncertain. A meta‐analysis of 45 empirical studies exploring the correlation between screen time and ADHD‐related behaviors revealed conflicting results, highlighting a limited amount of research in this field (Nikkelen et al. 2014). A study conducted in India reported a prevalence of smartphone addiction ranging from 39% to 44% among teenagers and young adults, emphasizing the significance of this issue (Davey and Davey 2014). Another study observed that 51.0% of the 506 participants displayed symptoms of smartphone addiction (Alageel et al. 2021). Excessive use of electronic devices has the potential to pose various challenges for students in their daily lives.

In technologically advanced societies, poor sleep quality has emerged as a significant public health concern (Cheung and Wong 2011). The increasing use of technology, including smartphones, has contributed to the growing prevalence of sleep difficulties (Maurya et al. 2022). Spending 3 or more hours per day engaged with screens has been associated with a notably higher risk of experiencing frequent sleep problems in early adulthood (Hysing et al. 2015). To promote overall health and development, the US National Sleep Foundation recommends that adolescents (aged 14–17 years) aim for 8–10 h of sleep while young adults (aged 18–25 years) should target approximately 7–9 h per night (NS Foundation n.d.). Insufficient or disrupted sleep has become a prevalent issue among youth and adolescents worldwide (Gradisar, Gardner, and Dohnt 2011). Several studies have shown that students who excessively use mobile phones, especially at night or past midnight instead of resting, face disruptions in their sleep and wake cycles. This disruption can impact melatonin levels and increase the likelihood of mental health problems, including depression, stress, anxiety, and social dysfunction (Thomée, Härenstam, and Hagberg 2011; Yogesh, Abha, and Priyanka 2014).

The primary objective of this study is to explore the connection between smartphone addiction, ADHD, and sleep problems among university students. Furthermore, it aims to provide policy recommendations that can support teenagers in improving their mental well‐being and academic performance by promoting limited screen time and adopting healthy sleep habits. In essence, this study seeks to investigate the association between smartphone screen time, sleep problems, and ADHD among young adults, with the intention of shedding light on these relationships.

The increasing prevalence of smartphone addiction among university students has become a significant concern, prompting a deeper investigation into its broader impacts on health and well‐being. This study hypothesizes that excessive smartphone use negatively affects sleep quality and exacerbates symptoms of ADHD among university students. The motivation for this study stems from the observed rise in smartphone dependency, particularly within the academic context, where students rely heavily on their devices for both educational and social purposes. Given the critical role of adequate sleep and attentive functioning in academic success and overall health, understanding the extent to which smartphone addiction influences these factors is essential. By exploring these correlations, the study aims to contribute valuable insights into the potential risks associated with smartphone overuse.

## 2 Materials and Methods

### 2.1 Participants

This study focused on students enrolled at Bingöl University during the academic year of 2022–2023. A stratified random sampling method was chosen for the study. No specific inclusion and exclusion criteria were determined for the participants. After preliminary information, the scales were administered to all who agreed to participate in the study. After the data were collected, those that were complete and error‐free were evaluated. After the initial assessments, data from 443 participants who were determined to be eligible were evaluated.

### 2.2 Procedure

In this cross‐sectional study, a sociodemographic data form comprising 13 questions was created by the researchers. The screen time of the students on their smartphones was asked through the digital balance and digital health applications in the settings. Alongside this form, questionnaires, including the Smartphone Addiction Scale‐Short Version (SAS‐SV), the Pittsburgh Sleep Quality Index (PSQI), and the Adult ADHD Self‐Report Scale (ASRS‐v1.1), were employed. The distribution of the questionnaires took place online through an internet platform, with participating students receiving a link to access them. It should be emphasized that student participation in the study was voluntary.

### 2.3 Measures

The SAS‐SV is a 10‐item, 6‐point Likert‐type scale developed to assess the risk of smartphone addiction (Kwon, Kim, et al., 2013). The scale is the short form of the SAS developed by Kwon, Lee, et al. (2013). The scale examines three aspects: “daily life disturbance” (three questions), “withdrawal” (four questions), and “cyberspace‐oriented relationship,” “overuse,” and “tolerance” (one question each). Each item is rated on a scale of 1–6, resulting in a total score range of 10–60. Higher scores indicate a higher risk of addiction. The scale is unidimensional and does not include subscales. Its reliability and validity have been established through translation into Turkish (Noyan et al. 2015). However, the scale does not have a specific cut‐off point, and therefore, it does not provide a conclusive determination of the presence or absence of smartphone addiction. In the Turkish language, SAS and SAS‐SV are the only available tests for assessing smartphone addiction.

Participants were asked to respond to a series of questions regarding their behavior over the past 6 months using the six‐item version of the ASRS‐v1.1, available in multiple languages at http://www.hcp.med.harvard.edu/ncs/asrs.php. The questionnaire comprises six items designed to assess levels of attention problems and hyperactivity. Four items focus on attention problems, while the remaining two items address hyperactivity. To calculate scores, the scoring method proposed by Kessler et al. was utilized, where each item is assigned a value based on the scale: never = 0, rarely = 1, sometimes = 2, often = 3, very often = 4. The scores for all six items were summed to generate a global score, with only the overall score being computed (Kessler et al. 2007).

Sleep quality was evaluated using the PSQI (Buysse et al. 1989), which assesses subjective sleep quality over the past month. The questionnaire consists of 19 self‐rated questions and 5 questions rated by the bed partner. The 19 items are categorized into seven components: subjective sleep quality, sleep latency, sleep duration, sleep efficiency, sleep disturbances, use of sleep medication, and daytime dysfunction. Component scores are then combined to calculate a global PSQI score ranging from 0 to 21, with higher scores indicating poorer sleep quality. PSQI scores above 5 were considered abnormal. The scale was adapted to Turkish culture by Ağargün, Kara, and Anlar (1996).

### 2.4 Statistical Analyses

All statistical evaluations of our current study were performed using version 22.0 of the Statistical Package for Social Sciences program (SPSS for Windows, version 22.0, SPSS, Chicago, IL, USA). The data obtained during the study were loaded into the SPSS 22.0 program as soon as it was obtained. Normally distributed continuous numerical data were compared with Student's *t*‐test. Categorical data were compared with the chi‐square test. Whether the data showed normal distribution or not was evaluated with the Shapiro–Wilk test. Pearson's correlation test was used to test whether there is a correlation between age, average sleep time, and scale scores of the SAS‐SV, PSQI, and ASRS‐v1.1. The effects of gender, age, alcohol, smoking, ASRS‐v1.1 scores, and PSQI scores on SAS‐SV scores were analyzed by linear regression test. We accepted an alpha value of 0.05 and below as statistical significance in all statistical analyses we performed.

## 3 Results

The total number of participants was 443. The mean age of the participants was 20.97 ± 3.29 and 72.7% (*n* = 322) were female. The most exposed screen for the majority of participants was the smartphone (94.8%, *n* = 420) and many used it for social media surfing (49.9%, *n* = 221). The sociodemographic data of the participants are summarized in Table 1.

**TABLE 1:** Sociodemographic characteristics of the participants.

	*n* (%)
Gender	
Male	121 (27.3%)
Female	322 (72.7%)
Class	
First	240 (54.2%)
Second	162 (36.6%)
Third and above	41 (9.2%)
Alcohol	
Yes	28 (6.3%)
No	415 (93.7%)
Smoking	
Yes	108 (24.4%)
No	335 (75.6%)
Exercise	
No	99 (22.3%)
Occasionally	304 (68.6%)
Regularly	40 (9%)
Screen preference	
Television	7 (1.6%)
Computer	14 (3.2%)
Smartphone	420 (94.8%)
Tablet phone	2 (0.5%)
Intended use of the screen	
To study	61 (13.8%)
Playing game	9 (2%)
Surfing social media	221 (49.9%)
Chatting on social media	67 (15.1%)
Watching TV series and movies	85 (19.2%)
Screen exposure time	
< 30 min	11 (2.5%)
30 min–2 h	78 (17.6%)
2–4 h	164 (37%)
4–8 h	151 (34.1%)
> 8 h	39 (8.8%)

The participants were asked how many times a day they check their phones, how many hours they use on average, which application they use the most, and whether they consider themselves to be smartphone addicts. Smartphone active screen time learned (via app). Smartphone on‐screen time reported via the app was found to be 25.1% for 4–6 h, 10.2% for 6–8 h, and 6.3% for more than 8 h. The data are summarized in Table 2.

**TABLE 2:** Behaviors and attitudes of the participants regarding the smartphone.

	*n* (%)
Average number of phone checks per day	
< 10 times	31 (7%)
10–20 times	122 (27.5%)
20–30 times	111 (25.1%)
30–40 times	62 (14%)
> 40 times	117 (26.4%)
Average hours of phone usage per day	
<1 h	8 (1.8%)
1–2 h	73 (16.5%)
3–4 h	154 (34.8%)
5–6 h	103 (23.3%)
6–7 h	52 (11.7%)
> 7 h	53 (12%)
Most preferred application on the phone	
Instagram	204 (46%)
WhatsApp	143 (32.3%)
YouTube	54 (12.2%)
Twitter	22 (5%)
Tiktok	7 (1.6%)
Other	13 (2.9%)
Do you consider yourself a smartphone addict?	
Not addicted	120 (27.1%)
No idea	56 (12.6%)
Perhaps	191 (43.1%)
Addicted	76 (17.2%)
Smartphone on screen time (via application)	
< 1 h	54 (12.2%)
1–2 h	81 (18.3%)
2–4 h	124 (28%)
4–6 h	111 (25.1%)
6–8 h	45 (10.2%)
> 8 h	28 (6.3%)

The effects of sociodemographic characteristics on sleep quality were examined. We found that smoking, using a smartphone as a screen preference, and long‐term screen exposure significantly impair sleep quality. The effects of all variables on sleep quality are presented in Table 3. When the effect of smartphone usage patterns on sleep quality was examined, it was found that the average number of checks per day and the most frequently used application did not significantly affect sleep quality. It was determined that the average daily smartphone usage time, addiction self‐assessment and active screen time obtained via application significantly affect sleep quality. The effect of smartphone usage patterns on sleep quality is presented in Table 4.

**TABLE 3:** Evaluation of the relationship between sociodemographic variables and sleep quality.

	PSQI ≤ 5 (good)	PSQI ≥ 6 (poor)	*p* value^a^
Gender			
Male	22 (18.2%)	99 (81.8%)	0.311
Female	46 (14.3%)	276 (85.7%)	
Class			
First	35 (14.6%)	205 (85.4%)	0.877
Second	26 (16.0%)	136 (84.0%)	
Third and above	7 (17.1%)	34 (82.9%)	
Smoking			
Yes	7 (6.5%)	101 (93.5%)	0.003
No	61 (18.2%))	274 (81.8%)	
Alcohol			
Yes	3 (10.7%)	25 (89.3%)	0.598
No	65 (15.7%)	350 (84.3%)	
Exercise			
Never	8 (8.1%)	91 (91.9%)	0.054
Occasionally	53 (17.4%)	251 (82.6%)	
Regularly	7 (17.5%)	33 (82.5%)	
Screen preference			
Smartphone	60 (14.3%)	360 (85.7%)	0.015
Others	8 (34.8%)	15 (65.2%)	
Intended use of the screen			
To study	18 (29.5%)	43 (70.5%)	0.026
Playing game	2 (22.2%)	7 (77.8%)	
Surfing social media	28 (12.7%)	193 (87.3%)	
Chatting on social media	11 (16.4%)	56 (83.6%)	
Watching TV series and movies	9 (10.6%)	76 (89.4%)	
Screen exposure time			
< 30 min	1 (9.1%)	10 (90.9%)	0.003
30 min–2 h	23 (29.5%)	55 (70.5%)	
2–4 h	25 (15.2%)	139 (84.8%)	
4–8 h	13 (8.6%)	138 (91.4%)	
> 8 h	6 (15.4%)	33 (84.6%)	

Abbreviation: PSQI: Pittsburgh Sleep Quality Index.

^a^ *χ* ^2^ test.

**TABLE 4:** The effect of smartphone usage patterns on sleep quality.

	PSQI ≤ 5 (good)	PSQI ≥ 6 (poor)	*p* value^a^
Average number of phone checks per day			
< 10 times	7 (22.6%)	24 (77.4%)	0.192
10–20 times	25 (20.5%)	97 (79.5%)	
20–30 times	16 (14.4%)	95 (85.6%)	
30–40 times	7 (11.3%)	55 (88.7%)	
> 40 times	13 (11.1%)	104 (88.9%)	
Average hours of phone usage per day			
< 1 h	2 (25.0%)	6 (75.0%)	0.003
1–2 h	22 (30.1%)	51 (69.9%)	
3–4 h	25 (16.2%)	129 (83.8%)	
5–6 h	8 (7.8%)	95 (92.2%)	
6–7 h	5 (9.6%)	47 (90.4%)	
> 7 h	6 (11.3%)	47 (88.7%)	
Most preferred application on the phone			
Instagram	32 (15.7%)	172 (84.3%)	0.853
WhatsApp	23 (16.1%)	120 (83.9%)	
Other (YouTube, Twitter, Tiktok)	13 (13.5%)	83 (86.5%)	
Do you consider yourself a smartphone addict?			
Not addicted	28 (23.3%)	92 (76.7%)	0.003
No idea	13 (23.2%)	43 (76.8%)	
Perhaps	20 (10.5%)	171 (89.5%)	
Addicted	7 (9.2%)	69 (90.8%)	
Smartphone on screen time (via application)			
< 1 h	9 (16.7%)	45 (83.3%)	0.032
1–2 h	19 (23.5%)	62 (76.5%)	
2–4 h	24 (19.4%)	100(80.6%)	
4–6 h	10 (9.0%)	101 (91.0%)	
6–8 h	3 (6.7%)	42 (93.3%)	
> 8 h	3 (10.7%)	25 (89.3%)	

Abbreviation: PSQI: Pittsburgh Sleep Quality Index.

^a^ *χ* ^2^ test.

It was obtained from the literature that the cut‐off point of SAS‐SV was 31 for men and 33 for women. While the frequency of smartphone addiction was 50.4% (*n* = 61) in males, this rate was 47.2% (*n* = 152) in females. When the effect of smartphone addiction on sleep quality was examined, it was found that the sleep quality of addicted participants was statistically significantly worse in both men and women. The findings are presented in Table 5. While the ASRS‐v1.1 score of the smartphone‐addicted group was 17.40 ± 4.00, it was 14.21 ± 3.43 in the non‐addicted group (*p* < 0.001).

**TABLE 5:** The relationship of smartphone addiction with sleep quality.

		Female			Male		
		PSQI ≤ 5 (good)	PSQI ≥ 6 (poor)	*p* ^a^	PSQI ≤ 5 (good)	PSQI ≥ 6 (poor)	*p* ^a^
Smartphone addiction	Yes	10 (6.6%)	142 (93.4%)	< 0.001	4 (6.6%)	57 (93.4%)	0.002
	No	36 (21.2%)	134 (78.8%)		18 (30.0%)	42 (70.0%)	

Abbreviation: PSQI: Pittsburgh Sleep Quality Index.

^a^ *χ* ^2^ test.

The correlation between age, average sleep time, and scale scores of the SAS‐SV, PSQI, and ASRS‐v1.1 was examined. A statistically significant negative correlation was found between age and SAS‐SV and ASRS‐v1.1 scores (respectively, *r* = −0.152, *p* = 0.001; *r* = −0.96, *p* = 0.04). A statistically significant positive correlation was found between PSQI scores and SAS‐SV and ASRS‐v1.1 scores (respectively, *r* = 0.286, *p* < 0.001; *r* = 0.361, *p* < 0.001). A statistically significant positive correlation was found between the SAS‐SV score and the ASRS‐v1.1 score (*r* = 0.528, *p* < 0.001). A statistically significant negative correlation was found between ASRS‐v1.1 scores and average sleep duration (*r* = −0.17, *p* < 0.001).

The effects of age, gender, ASRS‐v1.1 scores, PSQI scores, alcohol use, and smoking on SAS‐SV scores were analyzed in a linear regression model. A statistically significant negative effect of age on SAS‐SV was found. ASRS‐v1.1 and PSQI scores showed a significant positive effect on SAS‐SV. The findings are presented in Table 6.

**TABLE 6:** Effect of variables on SAS‐SV scores.

				95.0% Confidence interval for *B*	
	*B*	Std. error	Significance	Lower bound	Upper bound
Constant	19.791	5.829	0.001	8.334	31.247
Gender	−0.916	1.084	0.399	−3.048	1.215
Age	−0.370	0.140	0.008	−0.645	−0.096
Alcohol use	0.653	2.039	0.749	−3.353	4.660
Smoking	−1.620	1.173	0.168	−3.926	0.686
ASRS‐v1.1	1.336	0.121	0.000	1.098	1.573
PSQI	0.332	0.157	0.035	0.024	0.640

Dependent variable: SAS–SV scores.

## 4 Discussion

Before starting the discussion, we would like to briefly summarize the study findings. In line with our study hypotheses, we found that smartphone addiction increased among young people. ADHD scores were greater and sleep quality was significantly worse among addicts, supporting our hypotheses that these variables would rise in tandem with increased addiction.

According to a study conducted by Kwon, Kim, et al. (2013), using cut‐off values of ≥ 31 for males and ≥ 33 for females to assess smartphone addiction, it was found that 16.9% of the 1519 participants exhibited smartphone addiction (Haug et al. 2015). However, other studies report even lower rates of smartphone addiction (S. M. Kim et al. 2014; S.‐G. Kim et al. 2019). The overall prevalence of smartphone addiction in a sample examined from these studies was 7.5%, with a higher rate observed in women than in men. In our study, the prevalence of smartphone addiction was determined as 50.4% (*n* = 61) in men and 47.2% (*n* = 152) in women. The fact that this rate was high in our study may depend on the time duration the study was conducted. The increase in smartphone use after COVID‐19 may have contributed to this rate. These findings are consistent with the results reported by Alageel et al. (2021). The higher prevalence of smartphone addiction among men may be attributed to their different patterns of smartphone usage, such as using smartphones more for work‐related activities, internet searches, and entertainment. On the other hand, women tend to spend more time on social network services or instant messaging, leading to increased smartphone usage (Bianchi and Phillips 2005; S.‐G. Kim et al. 2019). In addition, the addiction to smartphones among women has also been influenced by the rise of online shopping activities.

The inconsistency in the current literature regarding the prevalence of smartphone addiction may stem from variations in study methodologies, sample characteristics, and geographical factors. To enhance the validity and comparability of findings, future research should employ a standardized assessment tool and consider regional variations (S.‐G. Kim et al. 2019). Within the scope of this study, a linear regression model was utilized to investigate the impact of age, ADHD, and sleep quality on smartphone addiction. The analysis demonstrated statistically significant associations between these factors and smartphone addiction.

In this study, a significant majority of participants, comprising 79.9%, reported being exposed to screens for more than 2 h per day. Among them, 42.9% reported screen exposure exceeding 4 h. Furthermore, 17.2% of participants self‐identified as being addicted to their smartphones. Notably, 49.9% of participants reported using their smartphones primarily for browsing social media platforms. An overwhelming majority of 94.8% stated that they used smartphones as their primary screen. The portability and easy accessibility of smartphones contribute to their potential for addictive behaviors.

Individuals with ADHD are known to have a higher vulnerability to addiction compared to those without ADHD. Research has indicated that individuals with smartphone addiction are more likely to develop symptoms of ADHD (S.‐G. Kim et al. 2019). A study conducted among university students also found a positive correlation between increasing levels of screen time exposure and a higher risk of self‐perceived attention problems and hyperactivity levels (Montagni, Guichard, and Kurth 2016). Similarly, in the present study, it was observed that smartphone addiction is associated with self‐perceived attention deficit and hyperactivity. Even after controlling for other variables, the presence of ADHD showed a significant association with smartphone addiction. Individuals with ADHD often exhibit impulsivity, difficulties in behavior control, and heightened sensitivity to rewards. The sense of control, immediate response, and the opportunity for self‐expression associated with smartphone use can provide strong motivation and rewards for adolescents with ADHD. Due to the instant response and rewards offered by smartphones, as well as their portability, adolescents with ADHD may find smartphone use more appealing than those without the condition. Deficits in self‐control and inhibition can contribute to increased difficulties with excessive smartphone use and the exacerbation of ADHD symptoms (S.‐G. Kim et al. 2019). Furthermore, Billieux et al. suggested that impulsive traits such as impatience, low perseverance, and the duration of cell phone possession are robust predictors of addiction (Billieux, Van der Linden, and Rochat 2008). The widespread adoption of smartphones in recent years has led to an increased susceptibility to smartphone addiction, which can be viewed as the convergence of preexisting issues related to mobile phones and IA (Haripriya, Samuel, and Megha 2019). Research has demonstrated similarities between smartphone addiction and IA (Ben‐Yehuda, Greenberg, and Weinstein 2016). Children with ADHD often exhibit deficits in attention, strategic flexibility, planning, working memory, and behavior regulation. The lack of behavioral control in individuals with ADHD can contribute to difficulties in self‐regulating internet use (Sergeant 2000). In addition, psychological tests, experimental studies, and neuroimaging research have indicated a potential biological basis for the higher association between IA and ADHD, specifically involving reward deficiency and dopaminergic systems (Yoo et al. 2004).

The use of smartphones has been associated with a range of positive effects, such as access to updated information and improved academic performance. However, it also carries negative consequences, including substance abuse and addiction, which can adversely affect individuals' social and personal lives (Argiansya et al. 2021; Karki et al. 2021). One significant negative impact is the disruption of sleep, leading to various problems among adolescents and young adults (Maurya et al. 2022). Several studies have explored the relationship between mobile phone use and sleep quality. For instance, a survey conducted among college students in China found that 9.8% reported poor sleep quality associated with excessive mobile phone use (Tao et al. 2017). In this study, it was observed that both male and female participants who were addicted to smartphones experienced significantly poorer sleep quality. The problematic use of smartphones has been attributed to time displacement, where excessive smartphone usage delays bedtime as individuals engage with media content, leading to arousal and hindering sleep initiation. In addition, the biological impact of smartphones emitting blue light, which suppresses melatonin production, further contributes to difficulties in falling asleep and experiencing restful sleep (Argiansya et al. 2021; Hysing et al. 2015; Kuss and Lopez‐Fernandez 2016). Another study conducted in the United States found a correlation between screen time on mass media platforms, such as reading news online and using social media, and an increased likelihood of having a shorter sleep duration (Twenge, Krizan, and Hisler 2017). Similarly, adolescents who engaged in excessive screen time behaviors were more prone to insufficient sleep compared to those who did not engage in such behaviors (Baiden, Tadeo, and Peters 2019).

Previous studies conducted with university students worldwide have consistently reported a high prevalence of poor sleep quality, which is in line with our own findings. The prevalence rates of poor sleep quality were documented as 59.4% in Lithuania (Preišegolavičiūtė, Leskauskas, and Adomaitienė 2010), 52.7% in Lebanon (Assaad et al. 2014), 60% in the United States (Lund et al. 2010), 63% in India (Haripriya, Samuel, and Megha 2019), and 61.5% in Brazil (Rique et al. 2014). In our study, 81.8% of men and 85.7% of women exhibited poor sleep quality. A study conducted in Turkey in 2018 reported a lower prevalence rate of 52.4% for poor sleep quality. The recent surge in smartphone usage, especially during the COVID‐19 pandemic, may have contributed to the elevated rates of poor sleep quality observed in our study. Research has shown an increase in internet use and smartphone addiction during the pandemic (Hosen et al. 2021; Saadeh et al. 2021; Serra et al. 2021).

This study found a high prevalence of smartphone addiction among university students, which was associated with poor sleep quality and higher ADHD symptoms. However, the cross‐sectional design of the study makes it difficult to establish causality, and self‐reported data may be subject to subjective bias. The sample of university students limits the generalizability of the findings to the wider population. Although the findings are consistent with similar studies, methodological differences, and regional factors may cause variations in the results. Future research should examine these relationships more comprehensively using longitudinal designs in larger and more diverse populations.

Future studies should use standardized assessment tools to enhance comparability across studies. Longitudinal studies are needed to determine the causal relationships between smartphone addiction, ADHD, and sleep quality. Research should include more diverse populations beyond university students to improve generalizability.

## 5 Conclusions

The study revealed that impaired sleep quality was linked to factors such as using a smartphone as the primary screen and prolonged screen exposure. In addition, smartphone addiction, with around 50.4% of males and 47.2% of females classified as addicts, was strongly associated with poorer sleep quality in both genders.

The study found that poorer sleep quality was positively associated with both higher smartphone addiction and increased ADHD symptom scores. In addition, a strong positive correlation was identified between greater smartphone addiction and more severe ADHD symptoms. Furthermore, individuals with higher levels of ADHD symptoms tended to have shorter sleep durations.

In conclusion, our study contributes to the existing literature by examining the associations between smartphone overuse, ADHD symptoms, and sleep quality in university students. Furthermore, our findings shed light on the prevalent sleep quality issues experienced by university students, emphasizing the need for interventions and increased awareness of this important health concern.

## Author Contributions

**Ibrahim Zeyrek**: conceptualization, investigation, writing–original draft, methodology, writing–review and editing, project administration. **Muhammed Fatih Tabara**: investigation, writing–original draft, methodology, validation, formal analysis, writing–review and editing, supervision. **Mahmut Çakan**: data curation, supervision.

## Ethics Statement

The present study was approved by the Bingol University Ethics Committee (23.12.2022‐33117789/044/89758), and the principles reported in the Declaration of Helsinki were strictly followed in all study procedures.

## Consent

Written informed consent was obtained from all subjects that they wanted to voluntarily participate in the study.

## Conflicts of Interest

The authors declare no conflicts of interest.

### Peer Review

The peer review history for this article is available at https://publons.com/publon/10.1002/brb3.70137.

## Data Availability

The data are not publicly available due to privacy or ethical restrictions. The data that support the findings of this study are available from the corresponding author upon reasonable request.
